# Targeting SWI/SNF Complexes in Cancer: Pharmacological Approaches and Implications

**DOI:** 10.3390/epigenomes8010007

**Published:** 2024-02-04

**Authors:** Megan R. Dreier, Jasmine Walia, Ivana L. de la Serna

**Affiliations:** Department of Cell and Cancer Biology, University of Toledo College of Medicine and Life Sciences, 3000 Arlington Ave, Toledo 43614, OH, USA; megan.dreier@utoledo.edu (M.R.D.); jasmine.walia@rockets.utoledo.edu (J.W.)

**Keywords:** SWI/SNF chromatin-remodeling complexes, cancer, allosteric ATPAse inhibitors, bromodomain inhibitors, PROTACs, epigenetics, transcription, DNA damage

## Abstract

SWI/SNF enzymes are heterogeneous multi-subunit complexes that utilize the energy from ATP hydrolysis to remodel chromatin structure, facilitating transcription, DNA replication, and repair. In mammalian cells, distinct sub-complexes, including cBAF, ncBAF, and PBAF exhibit varying subunit compositions and have different genomic functions. Alterations in the SWI/SNF complex and sub-complex functions are a prominent feature in cancer, making them attractive targets for therapeutic intervention. Current strategies in cancer therapeutics involve the use of pharmacological agents designed to bind and disrupt the activity of SWI/SNF complexes or specific sub-complexes. Inhibitors targeting the catalytic subunits, SMARCA4/2, and small molecules binding SWI/SNF bromodomains are the primary approaches for suppressing SWI/SNF function. Proteolysis-targeting chimeras (PROTACs) were generated by the covalent linkage of the bromodomain or ATPase-binding ligand to an E3 ligase-binding moiety. This engineered connection promotes the degradation of specific SWI/SNF subunits, enhancing and extending the impact of this pharmacological intervention in some cases. Extensive preclinical studies have underscored the therapeutic potential of these drugs across diverse cancer types. Encouragingly, some of these agents have progressed from preclinical research to clinical trials, indicating a promising stride toward the development of effective cancer therapeutics targeting SWI/SNF complex and sub-complex functions.

## 1. SWI/SNF Structure and Function

**Figure 1:**
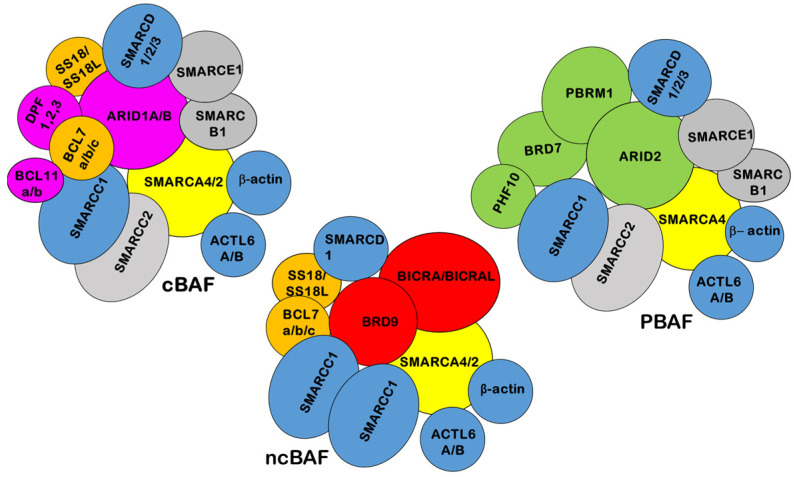
SWI/SNF Chromatin-Remodeling Complexes. Different SWI/SNF complexes exist in the form of cBAF, PBAF, and ncBAF. Yellow: SMARCA4 and SMARCA2 catalytic subunits. Purple: cBAF specific subunits. Green: PBAF specific subunits. Red: ncBAF specific subunits. Blue: subunits that assemble into all three complexes. Grey: subunits that assemble into cBAF and PBAF. Orange: subunits that assemble into cBAF and ncBAF.

SWI/SNF chromatin-remodeling enzymes are multi-subunit complexes, conserved across different eukaryotes, including fungi, plants, and animals [1]. Mammalian SWI/SNF is composed of a central catalytic subunit with ATPase activity that is either SMARCA4 (BRG1) or SMARCA2 (BRM) and 8–15 associated subunits (BAFs) [2]. Distinct SWI/SNF complexes have been purified from mammalian cells and designated as canonical (c)BAF, non-canonical (nc) BAF, or PBAF depending on the identity of the catalytic subunit and BAF composition [3,4,5,6,7,8,9] (Figure 1). In addition, mammalian complexes can vary in composition and in a cell-specific manner [10,11]. 

SWI/SNF remodels chromatin structures by using the energy from ATP hydrolysis. This results in DNA translocation around the nucleosome, making nucleosomal DNA accessible to nuclear factors [12,13,14]. SWI/SNF can slide or evict nucleosomes and disrupt the association of transcription factors from chromatin [15,16]. In vitro, either ATPase, SMARCA4, or SMARCA2, is capable of remodeling nucleosomes, and this remodeling activity is enhanced by a core complex containing SMARCB1 (INI1, BAF47), SMARCC1 (BAF155), and SMARCC2 (BAF170) [17]. The C-terminus of SMARCB1 interacts with an acidic patch of the nucleosome, securing SWI/SNF to the nucleosome, thereby promoting optimum chromatin-remodeling activity [18,19,20], while SMARCC1/2 assemble as dimers to provide a scaffold needed for proper SWI/SNF assembly [21]. 

Additional subunits provide features for optimal chromatin remodeling and impart structural specificity to the different SWI/SNF complexes. Both cBAF and PBAF complexes have a modular structure such that multiple subunits work together to interact with chromatin [6,20,22,23]. The largest subunit of cBAF complexes, ARID1A (and likely its paralog, ARID1B), serves as an assembly center for other BAF subunits and enhances nucleosome sliding activity in vitro [20]. ARID2 acts as a scaffold for the assembly of PBAF unique subunits, imparting architectural features that are distinct from BAF complexes as well as unique contacts with histone tails and nucleosomal DNA [22,23]. Actin-related proteins stabilize SWI/SNF complex formation and couple DNA translocation with ATP hydrolysis [24,25]. PBRM1, BRD7, and BRD9, as well as the catalytic subunits, SMARCA4 and SMARCA2, have bromodomains which bind acetylated histones, anchoring SWI/SNF to acetylated genomic sites [26,27,28,29]. Other BAFs such as SMARCD1, 2, and 3, and SMARCE1 are important for mediating interactions with a diversity of nuclear factors that recruit SWI/SNF to specific genomic sites [30,31,32,33,34,35,36,37,38]. Due to the distinctive and shared composition of BAFs, the various SWI/SNF complexes not only shape chromatin in a highly regulated manner, but also exhibit specialized functions in vivo. This high level of SWI/SNF regulation is frequently disrupted in cancer by mutations, altered subunit expression, activity, or aberrant SWI/SNF recruitment to genomic sites.

All three major SWI/SNF complexes play a critical role in the regulation of gene expression. cBAF, PBAF, and ncBAF are required for transcription, especially for the activation and repression of gene expression, during embryonic development and cellular differentiation, and in response to a plethora of external stimuli [8,39,40,41,42,43,44,45]. The various SWI/SNF complexes are targeted to different genomic regions to regulate overlapping and distinct genes and can compensate or have antagonistic roles. For example, SMARCA4 and SMARCA2 are paralogous subunits that exist in different SWI/SNF complexes and can compensate for each other in RB signaling but have antagonistic roles in p53 signaling [46,47]. ARID1B can partially compensate for ARID1A loss in the regulation of enhancer architecture in colorectal cancer cells [48]. The PBAF and cBAF complexes have divergent roles in the regulation of exhausted T cell differentiation; PBAF preserves stemness, while cBAF promotes differentiation to effector T cells [49]. Deficiency of the PBAF subunit, PBRM1, constricts T cell exhaustion and has been associated with an improved response to immunotherapy in melanoma and clear cell renal cell carcinoma [50,51,52]. Some of the distinct functions of the various complexes arise as a result of the genomic locations they occupy. cBAF complex occupancy is concentrated at gene enhancers [53,54], PBAF complexes at gene promoters [55], and ncBAF complexes at promoters and CTCF sites, which define topologically associated domain (TAD) boundaries [5]. Despite differential genome localization, there appears to be cross talk between the different complexes that is relevant in cancer. For example, the loss of PBAF results in the genomic re-distribution of cBAF complexes in melanoma cells, and the loss of cBAF disturbs the genomic occupancy of PBAF in transformed epithelial cells [55,56]. There is also the de-stabilization of cBAF complexes by the loss of SMARCE1 deficiency in clear cell meningioma which increases ncBAF activity [57]. SWI/SNF plays critical roles in transcription by regulating access to the transcriptional machinery, maintaining chromatin architecture in order to establish enhancer–promoter contacts [58]. Blocking SWI/SNF activity can have immediate consequences on genome-wide chromatin accessibility, transcription factor binding, and gene expression, indicating that SWI/SNF activity is continuously required to maintain proper chromatin architecture [59,60,61]. Because of its critical role in transcription, perturbations in the SWI/SNF complex structures and functions result in widespread gene expression alterations that promote tumorigenesis. 

SWI/SNF complexes play an important role in the maintenance of genome integrity and cellular survival from DNA damage. In response to double-strand breaks (DSBs), SWI/SNF complexes silence transcriptionally active genomic sites to facilitate efficient repair by either homologous recombination (HR) or non-homologous end joining (NHEJ). In HR, the PBAF and ncBAF complexes evict RNA polymerase II near the sites of DNA damage to rapidly silence transcription, while the cBAF complex maintains transcriptional silencing and promotes recruitment of the repair proteins, RNaseH1 and RAD52, to facilitate R loop resolution [62]. The BRD9 component of ncBAF also binds to an acetylated lysine on RAD54 and promotes RAD54 and RAD51 interactions [63]. PBAF also mediates transcriptional silencing and cBAF allows for recruitment of repair proteins during NHEJ [64,65]. SWI/SNF complexes have also been implicated in nucleotide excision repair (NER) through both the transcription-coupled repair (TCR) pathway and global genome repair (GGR) [66]. SWI/SNF increases NER efficiency on nucleosomal DNA by making it accessible to DNA repair enzymes [67,68] and has been reported to recruit NER factors, including XPC, ATM, XPG, PCNA, ERCC1, and ERCC5 in cells [69,70,71]. SWI/SNF increases base excision repair (BER) of nucleosomal DNA in vitro [72]. ARID1A-deficient cells accumulate abasic (AP) sites, exhibit delayed recruitment of BER repair proteins, and are highly sensitive to the combination of temozolomide and PARP inhibitors [73]. ARID1A promotes mismatch repair (MMR) by recruiting MSH2 to chromatin during DNA replication. A deficiency in SWI/SNF subunits is associated with defective mismatch repair and enhanced mutation load, rendering ARID1A-deficient tumors responsive to immune check point inhibitors [74,75,76]. Conversely, mismatch repair enzymes also interact with SMARCA4 to rewire the chromatin landscape in a manner that is conducive to tumorigenesis [77]. The SMARCA4 subunit is required for replication fork progression [78], and both SMARCA4 and ARID1A contribute to the resolution of transcription–replication conflicts which can lead to replication stress [79,80]. SWI/SNF deficiency in lung cancer cells results in increased origin firing, increased replication stress, and heightened sensitivity to ATR inhibitors [81]. In combination, these findings point to an important role for SWI/SNF in maintaining genome integrity and survival in response to DNA damage. 

## 2. SWI/SNF in Cancer

Approximately 20% of human cancers have mutations in SWI/SNF genes, many of which are loss-of-function mutations, suggesting that the disruption of SWI/SNF activity promotes cancer initiation or progression [82,83]. Indeed, some SWI/SNF subunits are verified tumor suppressors (see [84,85,86,87] for detailed recent reviews on SWI/SNF loss in cancer). The loss of ARID1A occurs in a variety of human cancers and de-regulates gene expression by disrupting SWI/SNF occupancy at enhancers, leading to the initiation of liver cancer [53,88]. The disruption of the SMARCB1 subunit frequently occurs in pediatric malignant rhabdoid tumors (MRT), epithelioid sarcomas, chordomas, and renal medullary carcinomas [89]. In the absence of SMARCB1, SWI/SNF binding to lineage-specific enhancers is disrupted while binding to oncogenic super-enhancers is retained, thus favoring tumorigenic gene expression [90]. The rescue of SMARCB1 in SMARCB1-deficient cancer cells abrogates proliferation and promotes differentiation [91,92]. The disruption of one allele of SMARCB1 in mice results in tumorigenesis with a loss of heterozygosity, which is consistent with the classical definition of a tumor suppressor [93,94]. The loss of ARID2 occurs frequently in hepatocellular carcinoma and melanoma [95,96] and disrupts PBAF complex assembly, also altering BAF genomic distribution to favor the expression of genes that promote invasion [55]. PBRM1 loss in renal clear cell carcinoma results in histone modifications at promoters, leads to genomic re-distribution of PBAF complexes, and promotes tumorigenic gene expression [97,98]. Many other studies indicate that alterations in the SWI/SNF complex occurring in cancer perturb enhancer dynamics, histone modifications, and transcription factor binding [48,99,100]. Hence, particular SWI/SNF subunits are tumor suppressors and their loss in cancer leads to the de-regulation of gene expression.

The SWI/SNF complex stands out as a highly promising target for cancer therapeutics. Strategies directed at targeting SWI/SNF encompass the inhibition of its catalytic activity or the suppression of specific subunit functions. The decision to inhibit the SWI/SNF complex, despite its recognized tumor-suppressive function, is rooted in a multifaceted rationale. SMARCA4 has a context-dependent pro-tumorigenic function. In hematological malignancies, SMARCA4 promotes transcription factor binding to lineage-specific *MYC* enhancer elements, driving long-range chromatin looping interactions with the *MYC* promoter to favor oncogenic gene expression [101]. Similarly, SMARCA4 is required to maintain chromatin accessibility at lineage-specific enhancers to favor binding of transcription factors that drive prostate cancer cell proliferation [102]. In several cancer types, SWI/SNF is also hijacked by oncogenes to elicit gene expression profiles that promote tumorigenesis [98,103,104,105,106,107]. The pharmacological inhibition of SWI/SNF function is useful in these cancer contexts. 

The inhibition of SWI/SNF function is also useful in cancers where there is a loss of a SWI/SNF subunit because residual SWI/SNF complexes can be rewired to take on oncogenic roles. Synthetic lethal relationships among SWI/SNF subunits render cancers with a loss-of-function mutation in one SWI/SNF subunit vulnerable to the inhibition of a second subunit. For example, SMARCA4-deficient cancer cells lose tumorigenicity and undergo cell cycle arrest and senescence upon the depletion of SMARCA2 [108,109,110]. ARID1A-deficient cancer cells are vulnerable to the loss of ARID1B, such that a concomitant loss of ARID1A and ARID1B disturbs enhancer dynamics at growth promoting loci [48]. Cancer cells lacking SMARCB1 or SMARCE1 demonstrate heightened ncBAF activity, leading to the induction of a growth-promoting gene expression signature. Consequently, these tumors exhibit a heightened susceptibility to BRD9 inhibition [9,57]. Therefore, there are multiple examples which show that SWI/SNF-deficient cancer cells can be targeted for elimination by further impairing SWI/SNF function.

SWI/SNF subunit loss can sensitize cancer cells to a wide range of therapeutics. There is a synthetic lethal relationship between SMARCA4 and the PTEN tumor suppressor in prostate cancer, rendering PTEN-deficient cells sensitive to SMARCA4 inhibition [111]. SMARCA4 loss sensitizes non-small cell lung cancer cells to CDK4/6 inhibition [112]. ARID1A loss in ovarian, breast, and pancreatic cancer cells sensitizes them to HDAC inhibitors [113,114,115]. SMARCA4-deficient lung and ovarian cancer cells are insensitive to HDAC inhibition but are highly sensitive to KDM6 histone demethylase inhibition [116]. The loss of other SWI/SNF subunits has been associated with an enhanced response to CDK4/6 inhibitors, immunotherapy, and other therapeutics [73,81,117]. In a recent Phase I clinical trial, durable responses to the ATR inhibitor, Ceralasertib, occurred in patients whose tumors were ARID1A-deficient and had high levels of DNA damage and inflammation [118]. In some contexts, SWI/SNF activity is required for proliferation and tumorigenesis [119], with high levels of SWI/SNF subunits associated with poorer patient survival [120,121]. Some missense mutations in SMARCA4 have been determined to be gain-of-function mutations, increasing remodeling efficiency and promoting promiscuous chromatin accessibility [25]. These observations have been the impetus to develop pharmacological approaches that intentionally inhibit SWI/SNF function in order to induce synthetic lethality or to exploit vulnerabilities that render tumors responsive to cancer therapeutics.

## 3. Pharmacological Inhibition of SWI/SNF Function

Several types of drugs are available to pharmacologically suppress SWI/SNF function (Table 1). 

**Figure 2:**
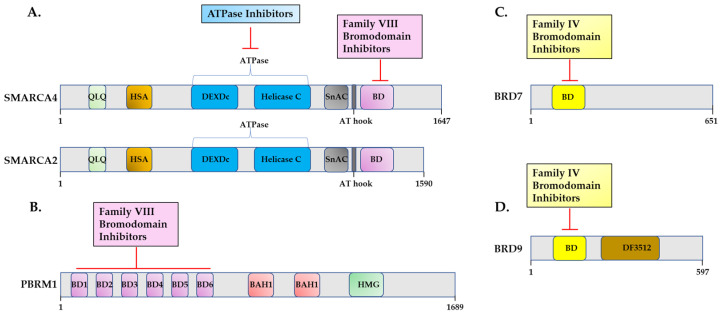
SWI/SNF subunits and the targeted domains. The most frequent approaches to inhibiting SWI/SNF function include the following: (**A**) Inhibition of the SMARCA4/SMARCA2 ATPase or inhibition of the SMARCA4 and SMARCA2 bromodomains with Family VIII inhibitors. (**B**) Family VIII inhibitors also bind to PBRM1 bromodomains. There are also several PBRM1-selective inhibitors. (**C**,**D**) Family IV bromodomain inhibitors bind to BRD7 and BRD9 bromodomains with varying selectivity for BRD9 or BRD7.

A unique approach that led to identification of cBAF-specific inhibitors involved screening molecules for the ability to de-repress the expression of the *BMI1* gene [122]. The binding of the macrolactam inhibitor, BD98, phenocopied some features of ARID1A/ARID1B loss and synergized with ATR inhibitors to kill a diverse array of cancer cells. The inhibition of ARID1A/ARID1B with BD98 in naïve CD8+T cells also improved the efficacy of adoptive T cell therapy by preventing differentiation [123]. Although BD98 is a promising drug, the most common approaches to pharmacologically suppress SWI/SNF function have been to use inhibitors of the SMARCA4/2 ATPases (Figure 2A) or with small molecules that bind Family VIII SWI/SNF bromodomains in SMARCA4, SMARCA2, PBRM1 (Figure 2A,B), as well as Family IV bromodomains in BRD7 and BRD9 (Figure 2C,D). 

The effectiveness of these small molecules can be extended or enhanced by constructing proteolysis-targeting chimeras (PROTACs) [124], which covalently link the bromodomain or ATPase-binding ligand to an E3 ligase-binding ligand to achieve degradation of particular SWI/SNF subunits. An ample number of preclinical studies on these drugs have provided evidence of their therapeutic potential. Some of the drugs have recently progressed to clinical trials (Table 2). 

### 3.1. Inhibitors of SWI/SNF Catalytic Activity

Active DNA-dependent ATPase A Domain inhibitor (ADAADi), generated by the activity of aminoglycoside phosphotransferases, was the first inhibitor of SWI/SNF catalytic activity to be discovered [130]. ADAADi competes with DNA for binding to DNA-dependent ATPase domains and potently inhibits SWI/SNF ATPase activity and ATP-dependent chromatin remodeling [131,132]. ADAADi was found to suppress prostate cancer tumor growth and to sensitize breast cancer cells to chemotherapeutics [133,134]. ADAADi also potently suppressed the proliferation of a number of other cancer cells, including HeLa, lung cancer, and hepatoblastoma cells. Although it is unclear how specific ADAADi is for SWI/SNF ATPases over that of other DNA-dependent ATPases, as the first SWI/SNF inhibitor, it has laid the foundation for subsequent advancements in this field. 

Allosteric inhibitors of the SMARCA4/SMARCA2 ATPase domains such as the compound, BRM014, are promising drugs for specific inhibition of SWI/SNF activity [135]. BRM014 and other ATPase inhibitors elicit very rapid changes in chromatin accessibility, occurring within minutes [59,60]. BRM014 potently inhibited the growth of an aggressive type of glioma harboring the histone H3 lysine 27 to a methionine mutation (H3K27M) in vitro and in vivo [136,137]. An ATPase degrader, JQ-dS-4, constructed by linking a BRG1 ATPase inhibitor to a phthalimide, which is a target for cereblon (CRBN) ubiquitin ligase, led to a reduction in chromatin accessibility at BRG1 binding sites and reduced the proliferation of H3K27M-driven gliomas. However, the degrader was less effective at curtailing tumor growth than the original ATPase inhibitor compound when used in an H3K27M glioma subcutaneous xenograft model, likely due to a reduced bioavailability of the drug. This study has demonstrated the potent anti-tumor effects of a SWI/SNF allosteric ATPase inhibitor on a highly aggressive brain cancer. It remains unclear whether generating a PROTAC from SWI/SNF ATPase inhibitors will be therapeutically advantageous over the unlinked molecule. Although promising, this study did not find that the drugs could cross the blood–brain barrier. This is a major challenge for translating these SWI/SNF inhibitors to the clinic for treating gliomas and other brain cancers. 

Acute myeloid leukemia (AML) cells were found to be highly sensitive to allosteric SMARCA4/SMARCA2 inhibitors, BRM011 and BRM014 [138]. Previous studies had determined that a subset of AML cells that are dependent on the MYC oncogene are sensitive to SMARCA4 depletion [101]. However, these allosteric ATPase compounds curtailed the proliferation of a wider range of leukemia cells. These cells were sensitive to the dual depletion of SMARCA4 and SMARCA2, but not to the depletion of SMARCA4 alone. BRM011 promoted both apoptosis and differentiation through the regulation of MYC and other oncogenic pathways, including mTORC1, IL2/STAT5, KRAS, and EMT pathways, curtailing tumor growth in AML xenograft models with no evidence of toxicity. The SMARCA4/SMARCA2 allosteric inhibitor, FHD-286, effectively suppressed the proliferation of a broad collection of patient-derived AML cells, promoted differentiation, and decreased tumor growth in vivo [125]. FHD-286 is currently in Phase I clinical trials as a monotherapy or combination therapy with the DNA-hypomethylating agent, decitabine, and low-dose antimetabolite, cytarabine, for patients with advanced hematological malignancies. 

Uveal melanoma is another cancer type that is highly sensitive to treatment with allosteric SMARCA4/SMARCA2 inhibitors [139]. This rare melanoma of the eye is dependent on the cooperation between the SWI/SNF complex and the melanocyte-inducing transcription factor (MITF) to drive a transcriptional program essential for uveal melanoma cell survival. Some uveal melanoma cell lines were highly sensitive to the depletion of SMARCA4 alone and others were only sensitive to the dual depletion of both SMARCA4 and SMARCA2. The treatment of uveal melanoma cells with BRM011 decreased chromatin accessibility at MITF binding sites and suppressed MITF and MITF target gene expression. Furthermore, BRM011 suppressed tumor growth in a uveal melanoma xenograft model, with no evidence of toxicity. The potential use of allosteric inhibitors of SMARCA4/SMARCA2 is a significant breakthrough for a cancer type that has few treatment options. Uveal melanoma is a highly aggressive cancer that metastasizes in half of all patients and, unlike cutaneous melanoma, does not respond to targeted and immunotherapy approaches [140]. The inhibition of SMARCA4/SMARCA2 with FHD-286 has advanced to Phase I clinical trials for patients with metastatic uveal melanoma [126].

### 3.2. Targeting SWI/SNF Bromodomains

An alternative approach toward inhibiting SWI/SNF function is with the use of bromodomain inhibitors that selectively bind any of the five SWI/SNF subunits bearing bromodomains [141]. Bromodomains are protein modules that bind acetylated lysines on histones and other proteins. They are important readers of the epigenetic code and can help recruit chromatin-remodeling complexes to regions containing acetylated chromatin [142]. In mammalian cells, the sixty-one different bromodomains are contained within forty-six proteins, all sharing a conserved structural motif consisting of a left-handed bundle of four α helices, linked by loop regions of variable length to form a hydrophobic pocket. Although the bromodomain is a conserved module, there are considerable sequence variations that impart specificity in the recognition of acetylated lysine motifs. The different bromodomains are classified into eight different families based on sequence and structural similarities. The enormous therapeutic potential of drugs, which selectively bind the extraterminal (BET) family of bromodomains in Family II, has stimulated interest in developing selective drugs that target other cancer-relevant bromodomains [143,144,145,146,147,148]. Drugs that selectively target Family VIII bromodomains, including SMARCA4, SMARCA2, and PBRM1, [141,149] as well as those which target Family IV, including BRD7 and BRD9 [150], disrupt SWI/SNF interactions with chromatin, and thereby exert genomic effects.

#### 3.2.1. Targeting SWI/SNF Family VIII Bromodomains

Several small molecules that bind Family VIII were developed and studied for their potential as cancer therapeutic agents. The first of these drugs to be characterized was PFI-3, which selectively binds to the bromodomains of SMARCA4, SMARCA2, and PBRM1 [151]. PFI-3-compromised embryonic stem cell maintenance and lineage specification, inhibited myogenesis and adipogenesis, and had mild effects on melanogenesis [152,153,154,155]. However, the inhibition with PFI-3 as a single drug had limited effects on breast cancer cell proliferation [156]. PFI-3 failed to completely displace SWI/SNF catalytic subunits from chromatin and did not re-capitulate synthetic lethality in SWI/SNF-deficient cancer cells as did the loss of the ATPase domain [157]. However, in multiple myeloma cells, PFI-3 displaced SMARCA2 and the oncogenic driver, NSD, from chromatin, suppressing oncogenic gene expression and tumor growth [158]. PFI-3 also enhanced the anti-cancer effects of temozolomide and sensitized highly resistant glioblastoma cells to the drug [159]. In combination with other DNA damaging drugs, PFI-3 induced death by displacing SMARCA4 from chromatin, compromising DSB repair [160]. These studies demonstrate that the anti-cancer effects of PFI-3 are contingent on whether the SWI/SNF VIII bromodomains are required for SWI/SNF recruitment to specific genomic sites that promote tumorigenesis or affect sensitivity to other chemotherapeutics. Furthermore, there is therapeutic potential in combining Family VIII bromodomain inhibitors with chemotherapeutics. Building upon PFI-3, next generation Family VIII bromodomain inhibitors were developed, which exert cellular effects [161,162]. Some of these newer drugs enhance cytotoxicity when combined with DNA-damaging agents in glioblastoma cells [161]. 

Family VIII inhibitors that are selective for PBRM1 bromodomains over those of SMARCA4 and SMARCA2 have also been developed and studied for anti-cancer effects [163,164,165]. Although a tumor suppressor in some cancers [166], PBRM1 promotes prostate cancer growth and progression [167,168]. As discussed previously, cancers with a loss of PBRM1 are more responsive to DNA damage and immune checkpoint inhibitors [50,51,52], suggesting that small molecule inhibitors selective for PBRM1 would be therapeutically useful. PBRM1-selective compounds were developed by targeting the second bromodomain of PBRM1 using an NMR-based fragment screen [164]. These drugs effectively inhibited the proliferation of an androgen receptor (AR)-positive prostate cancer cell line, with the most potent compound, PB16, exerting its effects in the sub-micromolar range. GNE-235 is a promising new PBRM1-selective inhibitor that has not yet been tested for its anti-tumor effects [165]. The promising results obtained with PB16 suggest that GNE-235 and other PBRM1-selective inhibitors should be further explored for anti-cancer effects as single drugs and in combination with DNA damaging agents and checkpoint inhibitors. 

The anti-cancer effects of Class VIII SWI/SNF bromodomain inhibitors can be enhanced by linking the bromodomain-binding ligand to an E3 ligase-binding ligand [102,165,169]. These PROTACs expand the anti-cancer effects of bromodomain inhibitors by degrading the bromodomain-containing subunits and taking them out of action. As a result, there is an inhibition of the whole subunit, not just the bromodomain. This strategy can displace SWI/SNF and suppress SWI/SNF activity at sites or in contexts that do not require SWI/SNF bromodomains for tumorigenesis. 

The PROTAC, ACBI1, is a Class VIII bromodomain-binding ligand [169] linked to a ligand of the von Hippel–Lindau (VHL) E3 ubiquitin ligase that induces rapid degradation of SMARCA4, SMARCA2, and PBRM1, altering the composition of both BAF and PBAF complexes. In contrast to the non-degrader form of ACBI1, which had no anti-cancer effect, the ACBI1 PROTAC killed SMARCA4-dependent leukemia cells as well as SWI/SNF-deficient cancer cells that are dependent on residual SWI/SNF activity. ACBI1 also compromised the growth of alveolar rhabdomyosarcoma tumors harboring the PAX3:FOX01 oncogene, demonstrating its anti-cancer effects in vivo [170]. 

AU-15330 is another SWI/SNF subunit degrader that similarly links a Class VIII bromodomain ligand to the VHL ligand to rapidly degrade SMARCA4, SMARCA2, and PBRM1 [102]. The treatment of AR-positive prostate cancer cells with AU-15330 reduced DNA accessibility at oncogenic enhancer elements and disrupted enhancer–promoter loops and AR and FOXA1 occupancy, thereby suppressing oncogenic gene expression. Changes in chromatin accessibility were also elicited by the concurrent genetic depletion of SMARCA4 and SMARCA2, indicating that the degradation of the catalytic subunits underlies the effects of AU-1530. AU-1530 suppressed tumor growth in xenograft models of prostate cancer and synergized with the AR antagonist, enzalutamide. AU-1530 was effective in other cancer types. The proliferation of MYC-driven multiple myeloma cells and estrogen receptor (ER)- and/or AR-positive breast cancer cells was also inhibited by AU-15330, with no toxicity to normal cells or to animals. H3K27M gliomas were highly sensitive to AU-15330 [171]. AU-15330 reduced chromatin accessibility at non-promoter sites, including the FOX01 enhancer. The loss of FOX01 expression led to the loss of oncogenic gene expression and suppression of glioma cell proliferation. The anti-cancer effects of AU-15330 were attributed to the loss of SMARCA4. SMARCA4 was found to maintain gliomas in an undifferentiated stem cell state, regulate genes involved in cell growth and extracellular matrix, and promote tumor growth in vivo in an H3K27M-dependent manner [136,137]. These studies demonstrate that AU-15330 suppresses SWI/SNF activity to exert anti-cancer effects on multiple cancer types driven by oncogenic enhancer circuitry. Furthermore, the synergy observed with the AU-15330 and enzalutamide combination provides compelling evidence for combining these drugs to enhance the effects of other therapeutics. 

A SMARCA2-selective degrader, A947, was generated by linking a Family IV bromodomain ligand to a moiety that is targeted to the von Hippel–Lindau (VHL) ubiquitin ligase complex [172]. Although the ligand could bind to SMARCA4, SMARCA2, and PBRM1 bromodomains, A947 rapidly degraded SMARCA2 with moderate selectivity over SMARCA4 and PBRM1. This change in ligand selectivity upon the generation of a PROTAC has been previously demonstrated and likely involves differential protein–protein interactions between the target proteins and the E3 ligase with some favoring degradation more than others [173]. Hence, the enhanced target selectivity that can be achieved with the proper design of the PROTAC demonstrates another advantage of using these molecules for inhibiting SWI/SNF bromodomains. A947 suppressed SMARCA4 mutant lung cancer growth in vivo and synergized with an inhibitor to the MCL1 anti-apoptotic protein to enhance apoptosis. PRT3789 is another potent and selective SMARCA2-targeted degrader that demonstrated synthetic lethality in SMARCA4-deficient cancers in vitro and in vivo and is currently in Phase I clinical trials for patients with SMARCA4 mutant solid tumors that express SMARCA2 [127].

#### 3.2.2. Targeting SWI/SNF Family IV Bromodomains

BRD9 and BRD7 are paralogous subunits with highly similar Family IV bromodomains [141], but are assembled into different SWI/SNF complexes (Figure 1). Loss-of-function mutations in the gene encoding BRD9 are rare; the BRD9 gene is amplified in multiple cancers [82] and is considered an oncogene in some of them. BRD9 is therefore an attractive target in cancer. The paralog, BRD7, has context-dependent tumor suppressive or tumor-promoting functions [174,175,176]. The depletion of BRD7 improves the response to the immune checkpoint and CHK1 inhibitors [52,177], suggesting that BRD7 is also a potential therapeutic target in some cancers. LP99 was the first drug to bind the BRD9 and BRD7 bromodomains, displacing both proteins from acetylated histones and inhibiting the proliferation of germ tumor cell lines, with EC50s in the micromolar range [178,179]. Drugs with higher potency and varying selectivity for either BRD9 and/or BRD7 have since been developed and tested for their anti-cancer effects [180]. 

Many inhibitors of the SWI/SNF Family IV bromodomains have focused on BRD9. BI-7271, BI-7273, and BI-9564 are potent selective BRD9 inhibitors that suppress leukemia cell proliferation [181]. AML cells depend on BRD9 to sustain the expression of the MYC oncogene and MYC transcriptional programs to prevent differentiation and promote proliferation. BI-7273 suppressed the MYC transcriptional program and proliferation in a BRD9 bromodomain-dependent manner, but did not affect proliferation of epithelial cancer cells [106]. I-BRD9, which has 700-fold selectivity over the BET family and 200-fold selectivity over BRD7 [182], also effectively suppressed leukemia cell proliferation [183]. I-BRD9 has been studied for its anti-cancer effects in a wide variety of cancers. I-BRD9 suppressed clear cell renal carcinoma tumor growth, a cancer in which BRD9 is up-regulated by FTO and recruited with SOX17 to enhancers that promote oncogenic gene expression [184]. I-BRD9 also blocked colorectal adenocarcinoma tumor growth through a mechanism that involves the regulation of glycolytic gene expression by BRD9 [185]. Natural phenols that inhibit BRD9 as well as I-BRD9 reduced colon cancer cell viability and colony formation by increasing DNA damage and apoptosis [186]. I-BRD9 sensitized ovarian cancer cells to DNA damaging agents, synergizing with PARP inhibitors to suppress ovarian cancer tumor growth [63]. TP-472, which has less selectivity for BRD9 over BRD7 compared to other BRD9 inhibitors, blocked melanoma tumorigenicity and tumor growth in vivo [121] as well as suppressed the proliferation of uterine leiomyosarcoma cells [187]. However, it is unclear whether BRD9 or BRD7, or both, were responsible for TP-472 anti-cancer effects. Another caveat is that TP-472 has been reported to have off-target effects on the efflux transporter ATP-binding cassette subfamily G member 2 (ABCG2) [188]. Therefore, although studies indicate that BRD9 inhibitors abrogate tumorigenicity in diverse cancer types, because the drugs differ in their selectivity, confirmation of the target via genetic depletion is crucial to understand drug mechanism in a particular cellular context.

Several BRD9 PROTACs have been constructed and shown to exert anti-cancer effects [189]. In an initial attempt to generate a BRD9 PROTAC, GSK39, a molecule with a structure similar to I-BRD9 was conjugated to a ligand that binds the cereblon (CRBN) ubiquitin ligase complex [182,190]. This compound was highly potent, but also degraded BRD7 and BET proteins, showing off-target effects. dBRD9 was then developed by utilizing BI-7273 to make the CRBN-targeting molecule. dBRD9 was highly selective for BRD9, did not degrade BRD7 or BET proteins in treated AML cells, and effectively blocked AML proliferation. dBRD9 suppressed oncogenic gene expression and induced synthetic lethality in synovial sarcoma cells more effectively than the parent compound, BI-7273, suggesting that a complete loss of the BRD9 protein is more deleterious than the sole inhibition of the bromodomain [5]. This is due, at least in part, to the bromodomain-independent binding of BRD9 to chromatin and the disruption of ncBAF when BRD9 expression is lost [191]. dBRD9 also suppressed multiple myeloma tumor growth by down-regulating ribosome biogenesis gene expression through a mechanism involving cooperation between BRD9 and BRD4, thereby increasing MYC transcriptional activity [192]. Clear cell meningioma cells with a loss of SMARCE1 were also highly sensitive to dBRD9 [57]. C6 is a recently developed orally active PROTAC that utilizes BI-7271 to make a CRBN-targeting molecule. This compound selectively degraded BRD9, did not demonstrate any observable degradation of BRD4 or BRD7, and potently suppressed AML growth in vitro and in vivo [193]. Other variations on BRD9 degraders include VZ-185, which degrades both BRD9 and BRD7 [194]. VZ-185 is VHL-based degrader that potently suppresses leukemia and malignant rhabdoid tumor cell proliferation. CFT8634 is a highly potent and orally bioavailable CRBN-based BRD9 degrader that is currently undergoing clinical trials for synovial or soft tissue sarcomas and SMARCB1-null tumors [129]. FHD-609 is a CRBN-based BRD9 degrader that is administered intravenously and is currently in Phase I clinical trials for patients with advanced synovial sarcoma or advanced SMARCB1-deficient tumors [128]. Hence, BRD9 inhibitors and degraders are promising drugs for the treatment of diverse cancers.

The first BRD7-selective inhibitors were recently developed and characterized [195]. The compounds 1-78 and 2-77 showed significantly increased affinity to the BRD7 bromodomain over that of BRD9 and were unable to displace BRD9 from chromatin, even at high concentrations. Both 1-78 and 2-77 inhibited AR target gene expression and cell growth in prostate cancer cells in a BRD7-dependent manner at sub-micromolar concentrations. In contrast, the BRD9 inhibitor, BI7273, had no effect on prostate cancer growth. These findings clearly demonstrate the selectivity of these compounds for BRD7. Interestingly, there was a high level of overlap in differentially regulated genes elicited via 2-77 and the SMARCA4/SMARCA2 degrader (ACBI1) as well as the PBRM1 inhibitor (PB16), and less overlap with the BRD9 degrader (dBRD9) and cBAF inhibitor (BD98), suggesting sub-complex specific regulation of gene regulation in prostate cancer cells. These promising findings on these novel BRD7-selective inhibitors deserve further exploration in other cancer types and in mouse models.

## 4. Conclusions

The pharmacological inhibition of SWI/SNF complexes is a novel approach to treat cancer. The most frequently employed approaches for inhibiting SWI/SNF function are to utilize allosteric inhibitors of the SWI/SNF ATPases or compounds that selectively bind to the SWI/SNF bromodomains. All of these methods exert anti-cancer effects in a context-dependent manner and are surprisingly non-toxic even when combined with other drugs. It is difficult to predict which approach is better in a particular context because of the limited number of studies that have compared these different approaches. Allosteric inhibitors of ATPase function offer the advantage of rapid and effective suppression of all SWI/SNF chromatin-remodeling activities. According to one study, making a PROTAC from allosteric inhibitor molecules does not increase anti-cancer effects in vitro and may decrease efficiency in vivo due to lower bioavailability [136]. This suggests that generating PROTACs from SWI/SNF allosteric inhibitors may not be advantageous. However, given that SMARCA4 and SMARCA2 exhibit functions independent of catalytic activity [196], the development of a bioavailable PROTAC from allosteric inhibitors may hold therapeutic potential and deserves further exploration. Several studies indicate that combining bromodomain ligands to degraders frequently generates more potent molecules that can also be more selective. These studies demonstrate that, in many cases, the PROTACs areadvantageous compared to the single-bromodomain ligands [169,172]. Despite the powerful effects of inhibiting bromodomain subunits, one study indicated that a disruption of SWI/SNF remodeling activity has a more profound effect on gene expression than the disruption of SWI/SNF sub-complexes through bromodomain inhibition and degradation [195]. However, the loss of ATPase subunits does not necessarily eliminate all SWI/SNF functions, as seen in cancers such as small cell carcinoma of the ovary—hypercalcemic type (SCCOHT)—where there is a dual loss of SMARCA4/2, but which retain residual complexes without the catalytic activity that may contribute to oncogenic gene expression [196].

While the chemical inhibition of SWI/SNF is a promising approach for treating many cancers, there are still unanswered questions and challenges to overcome. Although gliomas, as well as many cancers that metastasize to the brain, are sensitive to SWI/SNF inhibition, the inability to cross the blood–brain barrier will deter their efficacy in these contexts. Furthermore, although not toxic, the long-term effects of drugs that inhibit SWI/SNF function are not known. Given its tumor suppressor role, a long-term pharmacological inhibition of SWI/SNF could predispose cancer patients to secondary malignancies. Extended SWI/SNF inhibition may also compromise adult stem cell maintenance, the heart, and tissues that require SWI/SNF activity. Studies on long-term exposure, potential development of drug resistance, and the identification of biomarkers that predict efficacy are among the issues that need to be further explored. 

## Figures and Tables

**Table 1:** Drugs that inhibit SWI/SNF function and their effects on cancer cells.

Drug Name	Mechanism of Action	Cancer Models in Which Drug Is Effective
ADAADi	Inhibitor of DNA-dependent ATPAse activity	Prostate, breast, hepatoblastoma, lung cancers, and HeLa cells
BRM014	Allosteric inhibitor of SMARCA4/2 catalytic activity	Glioma, leukemia
BRM011	Allosteric inhibitor of SMARCA4/2 catalytic activity	Leukemia
JQ-dS-4	PROTAC: CRBN-based allosteric inhibitor of SMARCA4/2 catalytic activity	Glioma
FHD-286	Allosteric inhibitor of SMARCA4/2 catalytic activity	Leukemia, uveal melanoma
PFI-3	Class VIII-selective SWI/SNF bromodomain inhibitor: SMARCA4/2, PBRM1	Multiple myeloma, glioblastoma when combined with temozolomide
PB16	PBRM1-selective bromodomain inhibitor	Prostate cancer
GNE-235	PBRM1-selective bromodomain inhibitor	Not yet tested
ACBI1	PROTAC: VHL-based Class VIII bromodomain-selective ligand that degrades SMARCA4/2 and PBRM1	Alveolar rhabdomyosarcoma, leukemia
AU15330	PROTAC: VHL-based Class VIII bromodomain-selective ligand that degrades SMARCA4/2 and PBRM1	Prostate, glioma, breast cancers, multiple myeloma, lymphoma, EWING sarcoma, SMARCA4-null melanoma, synergistic with enzalutamide in prostate cancer
A947	PROTAC: VHL-based Class VIII bromodomain-selective ligand that selectively degrades SMARCA2	Lung cancer
LP99	Class IV bromodomain inhibitor that inhibits BRD9 and BRD7	Germ tumor cells
BI-7271	Class IV bromodomain inhibitor that selectively inhibits BRD9	Leukemia
BI-7273	Class IV bromodomain inhibitor that selectively inhibits BRD9	Leukemia
BI-9564	Class IV bromodomain inhibitor that selectively inhibits BRD9	Leukemia
I-BRD9	Class IV bromodomain inhibitor that selectively inhibits BRD9	Leukemia, clear cell renal carcinoma, colorectal cancer, ovarian cancer in combination with DNA damaging agents
T-472	Class IV bromodomain inhibitor that selectively inhibits BRD9	Melanoma and uterine leiomyosarcoma
dBRD9	PROTAC: CRBN-based Class VIII bromodomain-selective ligand (uses BI7273) that degrades BRD9	Leukemia, synovial sarcoma, multiple myeloma, clear cell meningioma
C6	PROTAC: CRBN-based Class VIII bromodomain-selective ligand (uses BI7271) that degrades BRD9	Leukemia
VZ-185	PROTAC: VHL-based Class VIII bromodomain-selective ligand that degrades BRD7 and BRD9	Leukemia, malignant rhabdoid tumor
CFT8634	PROTAC: CRBN-based Class VIII bromodomain -elective ligand that degrades BRD9	Synovial sarcoma and soft-tissue sarcomas null for SMARCB1
FHD-609	PROTAC: CRBN-based Class VIII bromodomain-selective ligand that degrades BRD9	Synovial sarcoma and soft-tissue sarcomas null for SMARCB1
I-78	Class IV bromodomain inhibitor that selectively inhibits BRD7	Prostate cancer
2-77	Class IV bromodomain inhibitor that selectively inhibits BRD7	Prostate cancer
BD98	cBAF inhibitor, binds to cBAF complexes, and de-represses BMI1	Colon and breast cancer cells when combined with ATR inhibitor

**Table 2:** Clinical trials that target SWI/SNF components (https://clinicaltrials.gov/). Accessed on 28 December 2023.

Study Title	NCT Number	Phase/Status	Conditions	Drug/Intervention:	Mechanism	References
FHD-286 as Monotherapy or Combination Therapy in Subjects With Advanced Hematologic Malignancies	NCT04891757	Phase 1/recruiting	Advanced hematologic malignancies like R/R AML, R/R MDS, and R/R CMML not in blast crisis	FHD-286, low-dose cytarabine, decitabine	Selective, oral inhibitor of SMARCA4/2	[125]
FHD-286 in Subjects With Metastatic Uveal Melanoma	NCT04879017	Phase 1/active, not recruiting	Metastatic uveal melanoma	FHD-286	Selective, oral inhibitor of SMARCA4/2	[126]
A Study of PRT3789 in Participants With Select Advanced or Metastatic Solid Tumors With a SMARCA4 Mutation	NCT05639751	Phase 1/recruiting	Advanced, recurrent, or metastatic solid tumor malignancies with loss of SMARCA4 due to truncating mutation and/or deletion without concomitant SMARCA2 mutation or loss of SMARCA2 protein expression	PRT3789	Smarca2-bromodomain- binding degrader	[127]
FHD-609 in Subjects With Advanced Synovial Sarcoma or Advanced SMARCB1-Loss Tumors	NCT04965753	Phase 1/active, not recruiting	Advanced synovial sarcoma or advanced SMARCB1-loss tumors	FHD-609	Intravenously administered agent that binds the BRD9 bromodomain and leads to degradation of the BRD9 protein	[128]
A Study to Assess the Safety and Tolerability of CFT8634 in Locally Advanced or Metastatic SMARCB1-Perturbed Cancers, Including Synovial Sarcoma and SMARCB1-Null Tumors	NCT05355753	Phase 1, Phase 2/active, not recruiting	Synovial or soft tissue sarcoma and SMARCB1-null tumors who have received prior systemic therapy, have relapsed/refractory tumors, have unresectable or metastatic disease, and are not candidates for available therapies known to confer clinical benefit	CFT8634	Orally taken agent that binds the BRD9 bromodomain and leads to BRD9 degradation	[129]

## References

[1] Hernandez-Garcia J., Diego-Martin B., Kuo P.H., Jami-Alahmadi Y., Vashisht A.A., Wohlschlegel J., Jacobsen S.E., Blazquez M.A., Gallego-Bartolome J. (2022). Comprehensive identification of SWI/SNF complex subunits underpins deep eukaryotic ancestry and reveals new plant components. Commun. Biol..

[2] Centore R.C., Sandoval G.J., Soares L.M.M., Kadoch C., Chan H.M. (2020). Mammalian SWI/SNF Chromatin Remodeling Complexes: Emerging Mechanisms and Therapeutic Strategies. Trends Genet..

[3] Yan Z., Cui K., Murray D.M., Ling C., Xue Y., Gerstein A., Parsons R., Zhao K., Wang W. (2005). PBAF chromatin-remodeling complex requires a novel specificity subunit, BAF200, to regulate expression of selective interferon-responsive genes. Genes Dev..

[4] Xue Y., Canman J.C., Lee C.S., Nie Z., Yang D., Moreno G.T., Young M.K., Salmon E.D., Wang W. (2000). The human SWI/SNF-B chromatin-remodeling complex is related to yeast rsc and localizes at kinetochores of mitotic chromosomes. Proc. Natl. Acad. Sci. USA.

[5] Michel B.C., D’Avino A.R., Cassel S.H., Mashtalir N., McKenzie Z.M., McBride M.J., Valencia A.M., Zhou Q., Bocker M., Soares L.M.M. (2018). A non-canonical SWI/SNF complex is a synthetic lethal target in cancers driven by BAF complex perturbation. Nat. Cell Biol..

[6] Mashtalir N., D’Avino A.R., Michel B.C., Luo J., Pan J., Otto J.E., Zullow H.J., McKenzie Z.M., Kubiak R.L., St Pierre R. (2018). Modular Organization and Assembly of SWI/SNF Family Chromatin Remodeling Complexes. Cell.

[7] Alpsoy A., Dykhuizen E.C. (2018). Glioma tumor suppressor candidate region gene 1 (GLTSCR1) and its paralog GLTSCR1-like form SWI/SNF chromatin remodeling subcomplexes. J. Biol. Chem..

[8] Gatchalian J., Malik S., Ho J., Lee D.S., Kelso T.W.R., Shokhirev M.N., Dixon J.R., Hargreaves D.C. (2018). A non-canonical BRD9-containing BAF chromatin remodeling complex regulates naive pluripotency in mouse embryonic stem cells. Nat. Commun..

[9] Wang X., Wang S., Troisi E.C., Howard T.P., Haswell J.R., Wolf B.K., Hawk W.H., Ramos P., Oberlick E.M., Tzvetkov E.P. (2019). BRD9 defines a SWI/SNF sub-complex and constitutes a specific vulnerability in malignant rhabdoid tumors. Nat. Commun..

[10] Olave I., Wang W., Xue Y., Kuo A., Crabtree G.R. (2002). Identification of a polymorphic, neuron-specific chromatin remodeling complex. Genes Dev..

[11] Soshnikova N.V., Azieva A.M., Klimenko N.S., Khamidullina A.I., Feoktistov A.V., Sheynov A.A., Brechalov A.V., Tatarskiy V.V., Georgieva S.G. (2023). A novel chromatin-remodeling complex variant, dcPBAF, is involved in maintaining transcription in differentiated neurons. Front. Cell Dev. Biol..

[12] Imbalzano A.N., Kwon H., Green M.R., Kingston R.E. (1994). Facilitated binding of TATA-binding protein to nucleosomal DNA. Nature.

[13] Kwon H., Imbalzano A.N., Khavari P.A., Kingston R.E., Green M.R. (1994). Nucleosome disruption and enhancement of activator binding by a human SW1/SNF complex. Nature.

[14] Narlikar G.J., Phelan M.L., Kingston R.E. (2001). Generation and interconversion of multiple distinct nucleosomal states as a mechanism for catalyzing chromatin fluidity. Mol. Cell.

[15] Dechassa M.L., Sabri A., Pondugula S., Kassabov S.R., Chatterjee N., Kladde M.P., Bartholomew B. (2010). SWI/SNF has intrinsic nucleosome disassembly activity that is dependent on adjacent nucleosomes. Mol. Cell.

[16] Li M., Hada A., Sen P., Olufemi L., Hall M.A., Smith B.Y., Forth S., McKnight J.N., Patel A., Bowman G.D. (2015). Dynamic regulation of transcription factors by nucleosome remodeling. eLife.

[17] Phelan M.L., Sif S., Narlikar G.J., Kingston R.E. (1999). Reconstitution of a core chromatin remodeling complex from SWI/SNF subunits. Mol. Cell.

[18] Valencia A.M., Collings C.K., Dao H.T., St Pierre R., Cheng Y.C., Huang J., Sun Z.Y., Seo H.S., Mashtalir N., Comstock D.E. (2019). Recurrent SMARCB1 Mutations Reveal a Nucleosome Acidic Patch Interaction Site That Potentiates mSWI/SNF Complex Chromatin Remodeling. Cell.

[19] Han Y., Reyes A.A., Malik S., He Y. (2020). Cryo-EM structure of SWI/SNF complex bound to a nucleosome. Nature.

[20] He S., Wu Z., Tian Y., Yu Z., Yu J., Wang X., Li J., Liu B., Xu Y. (2020). Structure of nucleosome-bound human BAF complex. Science.

[21] Chen J., Archer T.K. (2005). Regulating SWI/SNF subunit levels via protein-protein interactions and proteasomal degradation: BAF155 and BAF170 limit expression of BAF57. Mol. Cell. Biol..

[22] Yuan J., Chen K., Zhang W., Chen Z. (2022). Structure of human chromatin-remodelling PBAF complex bound to a nucleosome. Nature.

[23] Wang L., Yu J., Yu Z., Wang Q., Li W., Ren Y., Chen Z., He S., Xu Y. (2022). Structure of nucleosome-bound human PBAF complex. Nat. Commun..

[24] Schubert H.L., Wittmeyer J., Kasten M.M., Hinata K., Rawling D.C., Heroux A., Cairns B.R., Hill C.P. (2013). Structure of an actin-related subcomplex of the SWI/SNF chromatin remodeler. Proc. Natl. Acad. Sci. USA.

[25] Clapier C.R., Verma N., Parnell T.J., Cairns B.R. (2020). Cancer-Associated Gain-of-Function Mutations Activate a SWI/SNF-Family Regulatory Hub. Mol. Cell.

[26] Morrison E.A., Sanchez J.C., Ronan J.L., Farrell D.P., Varzavand K., Johnson J.K., Gu B.X., Crabtree G.R., Musselman C.A. (2017). DNA binding drives the association of BRG1/hBRM bromodomains with nucleosomes. Nat. Commun..

[27] Porter E.G., Dykhuizen E.C. (2017). Individual Bromodomains of Polybromo-1 Contribute to Chromatin Association and Tumor Suppression in Clear Cell Renal Carcinoma. J. Biol. Chem..

[28] Peng C., Zhou J., Liu H.Y., Zhou M., Wang L.L., Zhang Q.H., Yang Y.X., Xiong W., Shen S.R., Li X.L. (2006). The transcriptional regulation role of BRD7 by binding to acetylated histone through bromodomain. J. Cell. Biochem..

[29] Flynn E.M., Huang O.W., Poy F., Oppikofer M., Bellon S.F., Tang Y., Cochran A.G. (2015). A Subset of Human Bromodomains Recognizes Butyryllysine and Crotonyllysine Histone Peptide Modifications. Structure.

[30] Ito T., Yamauchi M., Nishina M., Yamamichi N., Mizutani T., Ui M., Murakami M., Iba H. (2001). Identification of SWI.SNF complex subunit BAF60a as a determinant of the transactivation potential of Fos/Jun dimers. J. Biol. Chem..

[31] Simone C., Forcales S.V., Hill D.A., Imbalzano A.N., Latella L., Puri P.L. (2004). p38 pathway targets SWI-SNF chromatin-remodeling complex to muscle-specific loci. Nat. Genet..

[32] Link K.A., Burd C.J., Williams E., Marshall T., Rosson G., Henry E., Weissman B., Knudsen K.E. (2005). BAF57 governs androgen receptor action and androgen-dependent proliferation through SWI/SNF. Mol. Cell. Biol..

[33] Priam P., Krasteva V., Rousseau P., D’Angelo G., Gaboury L., Sauvageau G., Lessard J.A. (2017). SMARCD2 subunit of SWI/SNF chromatin-remodeling complexes mediates granulopoiesis through a CEBPvarepsilon dependent mechanism. Nat. Genet..

[34] Witzel M., Petersheim D., Fan Y., Bahrami E., Racek T., Rohlfs M., Puchalka J., Mertes C., Gagneur J., Ziegenhain C. (2017). Chromatin-remodeling factor SMARCD2 regulates transcriptional networks controlling differentiation of neutrophil granulocytes. Nat. Genet..

[35] Aras S., Saladi S.V., Basuroy T., Marathe H.G., Lores P., de la Serna I.L. (2019). BAF60A mediates interactions between the microphthalmia-associated transcription factor and the BRG1-containing SWI/SNF complex during melanocyte differentiation. J. Cell. Physiol..

[36] Oh J., Sohn D.H., Ko M., Chung H., Jeon S.H., Seong R.H. (2008). BAF60a interacts with p53 to recruit the SWI/SNF complex. J. Biol. Chem..

[37] Hu X., Liu R., Hou J., Peng W., Wan S., Xu M., Li Y., Zhang G., Zhai X., Liang P. (2022). SMARCE1 promotes neuroblastoma tumorigenesis through assisting MYCN-mediated transcriptional activation. Oncogene.

[38] Feng J., Xu X., Fan X., Yi Q., Tang L. (2021). BAF57/SMARCE1 Interacting with Splicing Factor SRSF1 Regulates Mechanical Stress-Induced Alternative Splicing of Cyclin D1. Genes.

[39] Singh A.P., Archer T.K. (2014). Analysis of the SWI/SNF chromatin-remodeling complex during early heart development and BAF250a repression cardiac gene transcription during P19 cell differentiation. Nucleic Acids Res..

[40] Wang X., Song C., Ye Y., Gu Y., Li X., Chen P., Leng D., Xiao J., Wu H., Xie S. (2023). BRD9-mediated control of the TGF-beta/Activin/Nodal pathway regulates self-renewal and differentiation of human embryonic stem cells and progression of cancer cells. Nucleic Acids Res..

[41] Chandler R.L., Magnuson T. (2016). The SWI/SNF BAF-A complex is essential for neural crest development. Dev. Biol..

[42] Menon D.U., Kirsanov O., Geyer C.B., Magnuson T. (2021). Mammalian SWI/SNF chromatin remodeler is essential for reductional meiosis in males. Nat. Commun..

[43] Yamashita N., Morimoto Y., Fushimi A., Ahmad R., Bhattacharya A., Daimon T., Haratake N., Inoue Y., Ishikawa S., Yamamoto M. (2023). MUC1-C Dictates PBRM1-Mediated Chronic Induction of Interferon Signaling, DNA Damage Resistance, and Immunosuppression in Triple-Negative Breast Cancer. Mol. Cancer Res..

[44] Padilla-Benavides T., Olea-Flores M., Sharma T., Syed S.A., Witwicka H., Zuniga-Eulogio M.D., Zhang K., Navarro-Tito N., Imbalzano A.N. (2023). Differential Contributions of mSWI/SNF Chromatin Remodeler Sub-Families to Myoblast Differentiation. Int. J. Mol. Sci..

[45] Alpsoy A., Utturkar S.M., Carter B.C., Dhiman A., Torregrosa-Allen S.E., Currie M.P., Elzey B.D., Dykhuizen E.C. (2021). BRD9 Is a Critical Regulator of Androgen Receptor Signaling and Prostate Cancer Progression. Cancer Res..

[46] Xu Y., Zhang J., Chen X. (2007). The activity of p53 is differentially regulated by Brm- and Brg1-containing SWI/SNF chromatin remodeling complexes. J. Biol. Chem..

[47] Reisman D.N., Strobeck M.W., Betz B.L., Sciariotta J., Funkhouser W., Murchardt C., Yaniv M., Sherman L.S., Knudsen E.S., Weissman B.E. (2002). Concomitant down-regulation of BRM and BRG1 in human tumor cell lines: Differential effects on RB-mediated growth arrest vs CD44 expression. Oncogene.

[48] Kelso T.W.R., Porter D.K., Amaral M.L., Shokhirev M.N., Benner C., Hargreaves D.C. (2017). Chromatin accessibility underlies synthetic lethality of SWI/SNF subunits in ARID1A-mutant cancers. eLife.

[49] Baxter A.E., Huang H., Giles J.R., Chen Z., Wu J.E., Drury S., Dalton K., Park S.L., Torres L., Simone B.W. (2023). The SWI/SNF chromatin remodeling complexes BAF and PBAF differentially regulate epigenetic transitions in exhausted CD8(+) T cells. Immunity.

[50] Kharel A., Shen J., Brown R., Chen Y., Nguyen C., Alson D., Bluemn T., Fan J., Gai K., Zhang B. (2023). Loss of PBAF promotes expansion and effector differentiation of CD8(+) T cells during chronic viral infection and cancer. Cell Rep..

[51] Miao D., Margolis C.A., Gao W., Voss M.H., Li W., Martini D.J., Norton C., Bosse D., Wankowicz S.M., Cullen D. (2018). Genomic correlates of response to immune checkpoint therapies in clear cell renal cell carcinoma. Science.

[52] Pan D., Kobayashi A., Jiang P., Ferrari de Andrade L., Tay R.E., Luoma A.M., Tsoucas D., Qiu X., Lim K., Rao P. (2018). A major chromatin regulator determines resistance of tumor cells to T cell-mediated killing. Science.

[53] Mathur R., Alver B.H., San Roman A.K., Wilson B.G., Wang X., Agoston A.T., Park P.J., Shivdasani R.A., Roberts C.W. (2017). ARID1A loss impairs enhancer-mediated gene regulation and drives colon cancer in mice. Nat. Genet..

[54] McDonald B., Chick B.Y., Ahmed N.S., Burns M., Ma S., Casillas E., Chen D., Mann T.H., O’Connor C., Hah N. (2023). Canonical BAF complex activity shapes the enhancer landscape that licenses CD8(+) T cell effector and memory fates. Immunity.

[55] Carcamo S., Nguyen C.B., Grossi E., Filipescu D., Alpsoy A., Dhiman A., Sun D., Narang S., Imig J., Martin T.C. (2022). Altered BAF occupancy and transcription factor dynamics in PBAF-deficient melanoma. Cell Rep..

[56] Wang Z., Chen K., Jia Y., Chuang J.C., Sun X., Lin Y.H., Celen C., Li L., Huang F., Liu X. (2020). Dual ARID1A/ARID1B loss leads to rapid carcinogenesis and disruptive redistribution of BAF complexes. Nat. Cancer.

[57] St Pierre R., Collings C.K., Same Guerra D.D., Widmer C.J., Bolonduro O., Mashtalir N., Sankar A., Liang Y., Bi W.L., Gerkes E.H. (2022). SMARCE1 deficiency generates a targetable mSWI/SNF dependency in clear cell meningioma. Nat. Genet..

[58] Ahmad K., Brahma S., Henikoff S. (2024). Epigenetic pioneering by SWI/SNF family remodelers. Mol. Cell.

[59] Schick S., Grosche S., Kohl K.E., Drpic D., Jaeger M.G., Marella N.C., Imrichova H., Lin J.G., Hofstatter G., Schuster M. (2021). Acute BAF perturbation causes immediate changes in chromatin accessibility. Nat. Genet..

[60] Iurlaro M., Stadler M.B., Masoni F., Jagani Z., Galli G.G., Schubeler D. (2021). Mammalian SWI/SNF continuously restores local accessibility to chromatin. Nat. Genet..

[61] Hargreaves D.C. (2021). Chromatin openness requires continuous SWI/SNF activity. Nat. Genet..

[62] Davo-Martinez C., Helfricht A., Ribeiro-Silva C., Raams A., Tresini M., Uruci S., van Cappellen W.A., Taneja N., Demmers J.A.A., Pines A. (2023). Different SWI/SNF complexes coordinately promote R-loop- and RAD52-dependent transcription-coupled homologous recombination. Nucleic Acids Res..

[63] Zhou Q., Huang J., Zhang C., Zhao F., Kim W., Tu X., Zhang Y., Nowsheen S., Zhu Q., Deng M. (2020). The bromodomain containing protein BRD-9 orchestrates RAD51-RAD54 complex formation and regulates homologous recombination-mediated repair. Nat. Commun..

[64] Kakarougkas A., Ismail A., Chambers A.L., Riballo E., Herbert A.D., Kunzel J., Lobrich M., Jeggo P.A., Downs J.A. (2014). Requirement for PBAF in transcriptional repression and repair at DNA breaks in actively transcribed regions of chromatin. Mol. Cell.

[65] Watanabe R., Ui A., Kanno S., Ogiwara H., Nagase T., Kohno T., Yasui A. (2014). SWI/SNF factors required for cellular resistance to DNA damage include ARID1A and ARID1B and show interdependent protein stability. Cancer Res..

[66] Lans H., Marteijn J.A., Schumacher B., Hoeijmakers J.H., Jansen G., Vermeulen W. (2010). Involvement of global genome repair, transcription coupled repair, and chromatin remodeling in UV DNA damage response changes during development. PLoS Genet..

[67] Hara R., Sancar A. (2002). The SWI/SNF chromatin-remodeling factor stimulates repair by human excision nuclease in the mononucleosome core particle. Mol. Cell. Biol..

[68] Gaillard H., Fitzgerald D.J., Smith C.L., Peterson C.L., Richmond T.J., Thoma F. (2003). Chromatin remodeling activities act on UV-damaged nucleosomes and modulate DNA damage accessibility to photolyase. J. Biol. Chem..

[69] Zhang L., Zhang Q., Jones K., Patel M., Gong F. (2009). The chromatin remodeling factor BRG1 stimulates nucleotide excision repair by facilitating recruitment of XPC to sites of DNA damage. Cell Cycle.

[70] Ray A., Mir S.N., Wani G., Zhao Q., Battu A., Zhu Q., Wang Q.E., Wani A.A. (2009). Human SNF5/INI1, a component of the human SWI/SNF chromatin remodeling complex, promotes nucleotide excision repair by influencing ATM recruitment and downstream H2AX phosphorylation. Mol. Cell. Biol..

[71] Kothandapani A., Gopalakrishnan K., Kahali B., Reisman D., Patrick S.M. (2012). Downregulation of SWI/SNF chromatin remodeling factor subunits modulates cisplatin cytotoxicity. Exp. Cell Res..

[72] Menoni H., Gasparutto D., Hamiche A., Cadet J., Dimitrov S., Bouvet P., Angelov D. (2007). ATP-dependent chromatin remodeling is required for base excision repair in conventional but not in variant H2A.Bbd nucleosomes. Mol. Cell. Biol..

[73] Yu Z.C., Li T., Tully E., Huang P., Chen C.N., Oberdoerffer P., Gaillard S., Shih I.M., Wang T.L. (2023). Temozolomide Sensitizes ARID1A-Mutated Cancers to PARP Inhibitors. Cancer Res..

[74] Shen J., Ju Z., Zhao W., Wang L., Peng Y., Ge Z., Nagel Z.D., Zou J., Wang C., Kapoor P. (2018). ARID1A deficiency promotes mutability and potentiates therapeutic antitumor immunity unleashed by immune checkpoint blockade. Nat. Med..

[75] Kim K.J., Jung H.Y., Oh M.H., Cho H., Lee J.H., Lee H.J., Jang S.H., Lee M.S. (2015). Loss of ARID1A Expression in Gastric Cancer: Correlation with Mismatch Repair Deficiency and Clinicopathologic Features. J. Gastric Cancer.

[76] Tsuruta S., Kohashi K., Yamada Y., Fujiwara M., Koga Y., Ihara E., Ogawa Y., Oki E., Nakamura M., Oda Y. (2020). Solid-type poorly differentiated adenocarcinoma of the stomach: Deficiency of mismatch repair and SWI/SNF complex. Cancer Sci..

[77] Nargund A.M., Xu C., Mandoli A., Okabe A., Chen G.B., Huang K.K., Sheng T., Yao X., Teo J.M.N., Sundar R. (2022). Chromatin Rewiring by Mismatch Repair Protein MSH2 Alters Cell Adhesion Pathways and Sensitivity to BET Inhibition in Gastric Cancer. Cancer Res..

[78] Cohen S.M., Chastain P.D., Rosson G.B., Groh B.S., Weissman B.E., Kaufman D.G., Bultman S.J. (2010). BRG1 co-localizes with DNA replication factors and is required for efficient replication fork progression. Nucleic Acids Res..

[79] Bayona-Feliu A., Barroso S., Munoz S., Aguilera A. (2021). The SWI/SNF chromatin remodeling complex helps resolve R-loop-mediated transcription-replication conflicts. Nat. Genet..

[80] Tsai S., Fournier L.A., Chang E.Y., Wells J.P., Minaker S.W., Zhu Y.D., Wang A.Y., Wang Y., Huntsman D.G., Stirling P.C. (2021). ARID1A regulates R-loop associated DNA replication stress. PLoS Genet..

[81] Gupta M., Concepcion C.P., Fahey C.G., Keshishian H., Bhutkar A., Brainson C.F., Sanchez-Rivera F.J., Pessina P., Kim J.Y., Simoneau A. (2020). BRG1 Loss Predisposes Lung Cancers to Replicative Stress and ATR Dependency. Cancer Res..

[82] Shain A.H., Pollack J.R. (2013). The spectrum of SWI/SNF mutations, ubiquitous in human cancers. PLoS ONE.

[83] Kadoch C., Hargreaves D.C., Hodges C., Elias L., Ho L., Ranish J., Crabtree G.R. (2013). Proteomic and bioinformatic analysis of mammalian SWI/SNF complexes identifies extensive roles in human malignancy. Nat. Genet..

[84] Nguyen V.T., Tessema M., Weissman B.E., Chen J., Wang G.G., Lu J. (2023). The SWI/SNF Complex: A Frequently Mutated Chromatin Remodeling Complex in Cancer. Epigenetics in Oncology.

[85] Li Z., Zhao J., Tang Y. (2023). Advances in the role of SWI/SNF complexes in tumours. J. Cell. Mol. Med..

[86] Wang L., Tang J. (2023). SWI/SNF complexes and cancers. Gene.

[87] Dreier M.R., de la Serna I.L. (2022). SWI/SNF Chromatin Remodeling Enzymes in Melanoma. Epigenomes.

[88] Sun X., Wang S.C., Wei Y., Luo X., Jia Y., Li L., Gopal P., Zhu M., Nassour I., Chuang J.C. (2017). Arid1a Has Context-Dependent Oncogenic and Tumor Suppressor Functions in Liver Cancer. Cancer Cell.

[89] Cooper G.W., Hong A.L. (2022). SMARCB1-Deficient Cancers: Novel Molecular Insights and Therapeutic Vulnerabilities. Cancers.

[90] Wang X., Lee R.S., Alver B.H., Haswell J.R., Wang S., Mieczkowski J., Drier Y., Gillespie S.M., Archer T.C., Wu J.N. (2017). SMARCB1-mediated SWI/SNF complex function is essential for enhancer regulation. Nat. Genet..

[91] Kia S.K., Gorski M.M., Giannakopoulos S., Verrijzer C.P. (2008). SWI/SNF mediates polycomb eviction and epigenetic reprogramming of the INK4b-ARF-INK4a locus. Mol. Cell. Biol..

[92] Walhart T.A., Vacca B., Hepperla A.J., Hamad S.H., Petrongelli J., Wang Y., McKean E.L., Moksa M., Cao Q., Yip S. (2023). SMARCB1 Loss in Poorly Differentiated Chordomas Drives Tumor Progression. Am. J. Pathol..

[93] Guidi C.J., Sands A.T., Zambrowicz B.P., Turner T.K., Demers D.A., Webster W., Smith T.W., Imbalzano A.N., Jones S.N. (2001). Disruption of Ini1 leads to peri-implantation lethality and tumorigenesis in mice. Mol. Cell. Biol..

[94] Roberts C.W., Galusha S.A., McMenamin M.E., Fletcher C.D., Orkin S.H. (2000). Haploinsufficiency of Snf5 (integrase interactor 1) predisposes to malignant rhabdoid tumors in mice. Proc. Natl. Acad. Sci. USA.

[95] Li M., Zhao H., Zhang X., Wood L.D., Anders R.A., Choti M.A., Pawlik T.M., Daniel H.D., Kannangai R., Offerhaus G.J. (2011). Inactivating mutations of the chromatin remodeling gene ARID2 in hepatocellular carcinoma. Nat. Genet..

[96] Hodis E., Watson I.R., Kryukov G.V., Arold S.T., Imielinski M., Theurillat J.P., Nickerson E., Auclair D., Li L., Place C. (2012). A landscape of driver mutations in melanoma. Cell.

[97] Schoenfeld D.A., Zhou R., Zairis S., Su W., Steinbach N., Mathur D., Bansal A., Zachem A.L., Tavarez B., Hasson D. (2022). Loss of PBRM1 Alters Promoter Histone Modifications and Activates ALDH1A1 to Drive Renal Cell Carcinoma. Mol. Cancer Res..

[98] Yao X., Hong J.H., Nargund A.M., Ng M.S.W., Heng H.L., Li Z., Guan P., Sugiura M., Chu P.L., Wang L.C. (2023). PBRM1-deficient PBAF complexes target aberrant genomic loci to activate the NF-kappaB pathway in clear cell renal cell carcinoma. Nat. Cell Biol..

[99] Alver B.H., Kim K.H., Lu P., Wang X., Manchester H.E., Wang W., Haswell J.R., Park P.J., Roberts C.W. (2017). The SWI/SNF chromatin remodelling complex is required for maintenance of lineage specific enhancers. Nat. Commun..

[100] Hodges H.C., Stanton B.Z., Cermakova K., Chang C.Y., Miller E.L., Kirkland J.G., Ku W.L., Veverka V., Zhao K., Crabtree G.R. (2018). Dominant-negative SMARCA4 mutants alter the accessibility landscape of tissue-unrestricted enhancers. Nat. Struct. Mol. Biol..

[101] Shi J., Whyte W.A., Zepeda-Mendoza C.J., Milazzo J.P., Shen C., Roe J.S., Minder J.L., Mercan F., Wang E., Eckersley-Maslin M.A. (2013). Role of SWI/SNF in acute leukemia maintenance and enhancer-mediated Myc regulation. Genes Dev..

[102] Xiao L., Parolia A., Qiao Y., Bawa P., Eyunni S., Mannan R., Carson S.E., Chang Y., Wang X., Zhang Y. (2022). Targeting SWI/SNF ATPases in enhancer-addicted prostate cancer. Nature.

[103] Wang X., Sansam C.G., Thom C.S., Metzger D., Evans J.A., Nguyen P.T., Roberts C.W. (2009). Oncogenesis caused by loss of the SNF5 tumor suppressor is dependent on activity of BRG1, the ATPase of the SWI/SNF chromatin remodeling complex. Cancer Res..

[104] McBride M.J., Pulice J.L., Beird H.C., Ingram D.R., D’Avino A.R., Shern J.F., Charville G.W., Hornick J.L., Nakayama R.T., Garcia-Rivera E.M. (2018). The SS18-SSX Fusion Oncoprotein Hijacks BAF Complex Targeting and Function to Drive Synovial Sarcoma. Cancer Cell.

[105] Kim E.J., Liu P., Zhang S., Donahue K., Wang Y., Schehr J.L., Wolfe S.K., Dickerson A., Lu L., Rui L. (2021). BAF155 methylation drives metastasis by hijacking super-enhancers and subverting anti-tumor immunity. Nucleic Acids Res..

[106] Hohmann A.F., Martin L.J., Minder J.L., Roe J.-S., Shi J., Steurer S., Bader G., McConnell D., Pearson M., Gerstberger T. (2016). Sensitivity and engineered resistance of myeloid leukemia cells to BRD9 inhibition. Nat. Chem. Biol..

[107] Wood C.D., Veenstra H., Khasnis S., Gunnell A., Webb H.M., Shannon-Lowe C., Andrews S., Osborne C.S., West M.J. (2016). MYC activation and BCL2L11 silencing by a tumour virus through the large-scale reconfiguration of enhancer-promoter hubs. eLife.

[108] Keenen B., Qi H., Saladi S., Yeung M., De La Serna I. (2010). Heterogeneous SWI/SNF chromatin remodeling complexes promote expression of microphthalmia-associated transcription factor target genes in melanoma. Oncogene.

[109] Hoffman G.R., Rahal R., Buxton F., Xiang K., McAllister G., Frias E., Bagdasarian L., Huber J., Lindeman A., Chen D. (2014). Functional epigenetics approach identifies BRM/SMARCA2 as a critical synthetic lethal target in BRG1-deficient cancers. Proc. Natl. Acad. Sci. USA.

[110] Wilson B.G., Helming K.C., Wang X., Kim Y., Vazquez F., Jagani Z., Hahn W.C., Roberts C.W. (2014). Residual complexes containing SMARCA2 (BRM) underlie the oncogenic drive of SMARCA4 (BRG1) mutation. Mol. Cell. Biol..

[111] Ding Y., Li N., Dong B., Guo W., Wei H., Chen Q., Yuan H., Han Y., Chang H., Kan S. (2019). Chromatin remodeling ATPase BRG1 and PTEN are synthetic lethal in prostate cancer. J. Clin. Investig..

[112] Xue Y., Meehan B., Fu Z., Wang X.Q.D., Fiset P.O., Rieker R., Levins C., Kong T., Zhu X., Morin G. (2019). SMARCA4 loss is synthetic lethal with CDK4/6 inhibition in non-small cell lung cancer. Nat. Commun..

[113] Fukumoto T., Park P.H., Wu S., Fatkhutdinov N., Karakashev S., Nacarelli T., Kossenkov A.V., Speicher D.W., Jean S., Zhang L. (2018). Repurposing Pan-HDAC Inhibitors for ARID1A-Mutated Ovarian Cancer. Cell Rep..

[114] Cheng X., Zhao J.X., Dong F., Cao X.C. (2021). ARID1A Mutation in Metastatic Breast Cancer: A Potential Therapeutic Target. Front. Oncol..

[115] Kuo T.L., Cheng K.H., Chen L.T., Hung W.C. (2023). ARID1A loss in pancreas leads to islet developmental defect and metabolic disturbance. iScience.

[116] Romero O.A., Vilarrubi A., Alburquerque-Bejar J.J., Gomez A., Andrades A., Trastulli D., Pros E., Setien F., Verdura S., Farre L. (2021). SMARCA4 deficient tumours are vulnerable to KDM6A/UTX and KDM6B/JMJD3 blockade. Nat. Commun..

[117] Xue Y., Meehan B., Macdonald E., Venneti S., Wang X.Q.D., Witkowski L., Jelinic P., Kong T., Martinez D., Morin G. (2019). CDK4/6 inhibitors target SMARCA4-determined cyclin D1 deficiency in hypercalcemic small cell carcinoma of the ovary. Nat. Commun..

[118] Dillon M.T., Guevara J., Mohammed K., Patin E.C., Smith S.A., Dean E., Jones G.N., Willis S.E., Petrone M., Silva C. (2023). Durable responses to ATR inhibition with ceralasertib in tumors with genomic defects and high inflammation. J. Clin. Investig..

[119] Jimenez C., Antonelli R., Nadal-Ribelles M., Devis-Jauregui L., Latorre P., Sole C., Masanas M., Molero-Valenzuela A., Soriano A., Sanchez de Toledo J. (2022). Structural disruption of BAF chromatin remodeller impairs neuroblastoma metastasis by reverting an invasiveness epigenomic program. Mol. Cancer.

[120] Peng L., Li J., Wu J., Xu B., Wang Z., Giamas G., Stebbing J., Yu Z. (2021). A Pan-Cancer Analysis of SMARCA4 Alterations in Human Cancers. Front. Immunol..

[121] Mason L.D., Chava S., Reddi K.K., Gupta R. (2021). The BRD9/7 Inhibitor TP-472 Blocks Melanoma Tumor Growth by Suppressing ECM-Mediated Oncogenic Signaling and Inducing Apoptosis. Cancers.

[122] Chory E.J., Kirkland J.G., Chang C.Y., D’Andrea V.D., Gourisankar S., Dykhuizen E.C., Crabtree G.R. (2020). Chemical Inhibitors of a Selective SWI/SNF Function Synergize with ATR Inhibition in Cancer Cell Killing. ACS Chem. Biol..

[123] Guo A., Huang H., Zhu Z., Chen M.J., Shi H., Yuan S., Sharma P., Connelly J.P., Liedmann S., Dhungana Y. (2022). cBAF complex components and MYC cooperate early in CD8(+) T cell fate. Nature.

[124] Chamberlain P.P., Hamann L.G. (2019). Development of targeted protein degradation therapeutics. Nat. Chem. Biol..

[125] Collins M., Thomsen A., Gartin A., Sandoval G.J., Adam A., Reilly S., Delestre L., Penard-Lacronique V., Fiskus W., Bhalla K. (2023). Abstract 2122: The dual BRM/BRG1 (SMARCA2/4) inhibitor FHD-286 induces differentiation in preclinical models of AML. Proceedings of the American Association for Cancer Research Annual Meeting 2023. Cancer Res..

[126] Hentemann M. (2022). Abstract ND14: Pharmacological profile and anti-tumor properties of FHD-286: A novel BAF inhibitor for the treatment of transcription factor-driven cancers. Proceedings of the American Association for Cancer Research Annual Meeting 2022. Cancer Res..

[127] Hulse M., Agarwal A., Wang M., Carter J., Sivakumar M., Vidal B., Brown J., Moore A., Grego A., Bhagwat N. (2022). Abstract 3263: Preclinical characterization of PRT3789, a potent and selective SMARCA2 targeted degrader. Proceedings of the American Association for Cancer Research Annual Meeting 2022. Cancer Res..

[128] Dominici C., Mayhew D., Adam A., Uzan F., Garbitt-Amaral V., Mikse O., Antonakos B., Ahmad H., Parikh S., Lin M.Y. (2023). Abstract A049: Investigation of FHD-609, a potent degrader of BRD9, in preclinical models of acute myeloid leukemia (AML). Proceedings of the AACR-NCI-EORTC Virtual International Conference on Molecular Targets and Cancer Therapeutics. Mol. Cancer Ther..

[129] Jackson K.L., Agafonov R.V., Carlson M.W., Chaturvedi P., Cocozziello D., Cole K., Deibler R., Eron S.J., Good A., Hart A.A. (2022). Abstract ND09: The discovery and characterization of CFT8634: A potent and selective degrader of BRD9 for the treatment of SMARCB1-perturbed cancers. Proceedings of the American Association for Cancer Research Annual Meeting 2022. Cancer Res..

[130] Dutta P., Tanti G.K., Sharma S., Goswami S.K., Komath S.S., Mayo M.W., Hockensmith J.W., Muthuswami R. (2012). Global epigenetic changes induced by SWI2/SNF2 inhibitors characterize neomycin-resistant mammalian cells. PLoS ONE.

[131] Muthuswami R., Mesner L.D., Wang D., Hill D.A., Imbalzano A.N., Hockensmith J.W. (2000). Phosphoaminoglycosides inhibit SWI2/SNF2 family DNA-dependent molecular motor domains. Biochemistry.

[132] Felle M., Exler J.H., Merkl R., Dachauer K., Brehm A., Grummt I., Langst G. (2010). DNA sequence encoded repression of rRNA gene transcription in chromatin. Nucleic Acids Res..

[133] Muthuswami R., Bailey L., Rakesh R., Imbalzano A.N., Nickerson J.A., Hockensmith J.W. (2019). BRG1 is a prognostic indicator and a potential therapeutic target for prostate cancer. J. Cell. Physiol..

[134] Rakesh R., Chanana U.B., Hussain S., Sharma S., Goel K., Bisht D., Patne K., Swer P.B., Hockensmith J.W., Muthuswami R. (2021). Altering mammalian transcription networking with ADAADi: An inhibitor of ATP-dependent chromatin remodeling. PLoS ONE.

[135] Papillon J.P.N., Nakajima K., Adair C.D., Hempel J., Jouk A.O., Karki R.G., Mathieu S., Mobitz H., Ntaganda R., Smith T. (2018). Discovery of Orally Active Inhibitors of Brahma Homolog (BRM)/SMARCA2 ATPase Activity for the Treatment of Brahma Related Gene 1 (BRG1)/SMARCA4-Mutant Cancers. J. Med. Chem..

[136] Panditharatna E., Marques J.G., Wang T., Trissal M.C., Liu I., Jiang L., Beck A., Groves A., Dharia N.V., Li D. (2022). BAF Complex Maintains Glioma Stem Cells in Pediatric H3K27M Glioma. Cancer Discov..

[137] Mo Y., Duan S., Zhang X., Hua X., Zhou H., Wei H.J., Watanabe J., McQuillan N., Su Z., Gu W. (2022). Epigenome Programming by H3.3K27M Mutation Creates a Dependence of Pediatric Glioma on SMARCA4. Cancer Discov..

[138] Rago F., Rodrigues L.U., Bonney M., Sprouffske K., Kurth E., Elliott G., Ambrose J., Aspesi P., Oborski J., Chen J.T. (2022). Exquisite Sensitivity to Dual BRG1/BRM ATPase Inhibitors Reveals Broad SWI/SNF Dependencies in Acute Myeloid Leukemia. Mol. Cancer Res..

[139] Rago F., Elliott G., Li A., Sprouffske K., Kerr G., Desplat A., Abramowski D., Chen J.T., Farsidjani A., Xiang K.X. (2020). The Discovery of SWI/SNF Chromatin Remodeling Activity as a Novel and Targetable Dependency in Uveal Melanoma. Mol. Cancer Ther..

[140] Smit K.N., Jager M.J., de Klein A., Kiliҫ E. (2020). Uveal melanoma: Towards a molecular understanding. Prog. Retin. Eye Res..

[141] Filippakopoulos P., Picaud S., Mangos M., Keates T., Lambert J.P., Barsyte-Lovejoy D., Felletar I., Volkmer R., Muller S., Pawson T. (2012). Histone recognition and large-scale structural analysis of the human bromodomain family. Cell.

[142] Horn P.J., Peterson C.L. (2001). The bromodomain: A regulator of ATP-dependent chromatin remodeling?. Front. Biosci..

[143] Filippakopoulos P., Picaud S., Fedorov O., Keller M., Wrobel M., Morgenstern O., Bracher F., Knapp S. (2012). Benzodiazepines and benzotriazepines as protein interaction inhibitors targeting bromodomains of the BET family. Bioorganic Med. Chem..

[144] Filippakopoulos P., Qi J., Picaud S., Shen Y., Smith W.B., Fedorov O., Morse E.M., Keates T., Hickman T.T., Felletar I. (2010). Selective inhibition of BET bromodomains. Nature.

[145] Fish P.V., Filippakopoulos P., Bish G., Brennan P.E., Bunnage M.E., Cook A.S., Federov O., Gerstenberger B.S., Jones H., Knapp S. (2012). Identification of a chemical probe for bromo and extra C-terminal bromodomain inhibition through optimization of a fragment-derived hit. J. Med. Chem..

[146] Muller S., Filippakopoulos P., Knapp S. (2011). Bromodomains as therapeutic targets. Expert Rev. Mol. Med..

[147] Picaud S., Da Costa D., Thanasopoulou A., Filippakopoulos P., Fish P.V., Philpott M., Fedorov O., Brennan P., Bunnage M.E., Owen D.R. (2013). PFI-1, a highly selective protein interaction inhibitor, targeting BET Bromodomains. Cancer Res..

[148] Clegg M.A., Tomkinson N.C.O., Prinjha R.K., Humphreys P.G. (2019). Advancements in the Development of non-BET Bromodomain Chemical Probes. ChemMedChem.

[149] Singh M., Popowicz G.M., Krajewski M., Holak T.A. (2007). Structural ramification for acetyl-lysine recognition by the bromodomain of human BRG1 protein, a central ATPase of the SWI/SNF remodeling complex. ChemBioChem.

[150] Lloyd J.T., Glass K.C. (2018). Biological function and histone recognition of family IV bromodomain-containing proteins. J. Cell. Physiol..

[151] Gerstenberger B.S., Trzupek J.D., Tallant C., Fedorov O., Filippakopoulos P., Brennan P.E., Fedele V., Martin S., Picaud S., Rogers C. (2016). Identification of a Chemical Probe for Family VIII Bromodomains through Optimization of a Fragment Hit. J. Med. Chem..

[152] Fedorov O., Castex J., Tallant C., Owen D.R., Martin S., Aldeghi M., Monteiro O., Filippakopoulos P., Picaud S., Trzupek J.D. (2015). Selective targeting of the BRG/PB1 bromodomains impairs embryonic and trophoblast stem cell maintenance. Sci. Adv..

[153] Sharma T., Olea-Flores M., Imbalzano A.N. (2023). Regulation of the Wnt signaling pathway during myogenesis by the mammalian SWI/SNF ATPase BRG1. Front. Cell Dev. Biol..

[154] Sharma T., Robinson D.C.L., Witwicka H., Dilworth F.J., Imbalzano A.N. (2021). The Bromodomains of the mammalian SWI/SNF (mSWI/SNF) ATPases Brahma (BRM) and Brahma Related Gene 1 (BRG1) promote chromatin interaction and are critical for skeletal muscle differentiation. Nucleic Acids Res..

[155] Basuroy T., Dreier M., Baum C., Blomquist T., Trumbly R., Filipp F.V., de la Serna I.L. (2022). Epigenetic and pharmacological control of pigmentation via Bromodomain Protein 9 (BRD9). Pigment. Cell Melanoma Res..

[156] Wu Q., Sharma S., Cui H., LeBlanc S.E., Zhang H., Muthuswami R., Nickerson J.A., Imbalzano A.N. (2016). Targeting the chromatin remodeling enzyme BRG1 increases the efficacy of chemotherapy drugs in breast cancer cells. Oncotarget.

[157] Vangamudi B., Paul T.A., Shah P.K., Kost-Alimova M., Nottebaum L., Shi X., Zhan Y., Leo E., Mahadeshwar H.S., Protopopov A. (2015). The SMARCA2/4 ATPase Domain Surpasses the Bromodomain as a Drug Target in SWI/SNF-Mutant Cancers: Insights from cDNA Rescue and PFI-3 Inhibitor Studies. Cancer Res..

[158] Chong P.S.Y., Chooi J.Y., Lim J.S.L., Toh S.H.M., Tan T.Z., Chng W.J. (2021). SMARCA2 Is a Novel Interactor of NSD2 and Regulates Prometastatic PTP4A3 through Chromatin Remodeling in t(4;14) Multiple Myeloma. Cancer Res..

[159] Yang C., Wang Y., Sims M.M., He Y., Miller D.D., Pfeffer L.M. (2021). Targeting the Bromodomain of BRG-1/BRM Subunit of the SWI/SNF Complex Increases the Anticancer Activity of Temozolomide in Glioblastoma. Pharmaceuticals.

[160] Lee D., Lee D.Y., Hwang Y.S., Seo H.R., Lee S.A., Kwon J. (2021). The Bromodomain Inhibitor PFI-3 Sensitizes Cancer Cells to DNA Damage by Targeting SWI/SNF. Mol. Cancer Res..

[161] Yang C., He Y., Wang Y., McKinnon P.J., Shahani V., Miller D.D., Pfeffer L.M. (2023). Next-generation bromodomain inhibitors of the SWI/SNF complex enhance DNA damage and cell death in glioblastoma. J. Cell. Mol. Med..

[162] Wanior M., Preuss F., Ni X., Kramer A., Mathea S., Gobel T., Heidenreich D., Simonyi S., Kahnt A.S., Joerger A.C. (2020). Pan-SMARCA/PB1 Bromodomain Inhibitors and Their Role in Regulating Adipogenesis. J. Med. Chem..

[163] Melin L., Gesner E., Attwell S., Kharenko O.A., van der Horst E.H., Hansen H.C., Gagnon A. (2021). Design and Synthesis of LM146, a Potent Inhibitor of PB1 with an Improved Selectivity Profile over SMARCA2. ACS Omega.

[164] Shishodia S., Nunez R., Strohmier B.P., Bursch K.L., Goetz C.J., Olp M.D., Jensen D.R., Fenske T.G., Ordonez-Rubiano S.C., Blau M.E. (2022). Selective and Cell-Active PBRM1 Bromodomain Inhibitors Discovered through NMR Fragment Screening. J. Med. Chem..

[165] Cochran A.G., Flynn M. (2023). GNE-235: A Lead Compound Selective for the Second Bromodomain of PBRM1. J. Med. Chem..

[166] Hopson S., Thompson M.J. (2017). BAF180: Its Roles in DNA Repair and Consequences in Cancer. ACS Chem. Biol..

[167] Mota S.T.S., Vecchi L., Zoia M.A.P., Oliveira F.M., Alves D.A., Dornelas B.C., Bezerra S.M., Andrade V.P., Maia Y.C.P., Neves A.F. (2019). New Insights into the Role of Polybromo-1 in Prostate Cancer. Int. J. Mol. Sci..

[168] Hagiwara M., Fushimi A., Yamashita N., Bhattacharya A., Rajabi H., Long M.D., Yasumizu Y., Oya M., Liu S., Kufe D. (2021). MUC1-C activates the PBAF chromatin remodeling complex in integrating redox balance with progression of human prostate cancer stem cells. Oncogene.

[169] Sutherell C.L., Tallant C., Monteiro O.P., Yapp C., Fuchs J.E., Fedorov O., Siejka P., Muller S., Knapp S., Brenton J.D. (2016). Identification and Development of 2,3-Dihydropyrrolo [1,2-a]quinazolin-5(1H)-one Inhibitors Targeting Bromodomains within the Switch/Sucrose Nonfermenting Complex. J. Med. Chem..

[170] Bharathy N., Cleary M.M., Kim J.A., Nagamori K., Crawford K.A., Wang E., Saha D., Settelmeyer T.P., Purohit R., Skopelitis D. (2022). SMARCA4 biology in alveolar rhabdomyosarcoma. Oncogene.

[171] Mota M., Sweha S.R., Pun M., Natarajan S.K., Ding Y., Chung C., Hawes D., Yang F., Judkins A.R., Samajdar S. (2023). Targeting SWI/SNF ATPases in H3.3K27M diffuse intrinsic pontine gliomas. Proc. Natl. Acad. Sci. USA.

[172] Cantley J., Ye X., Rousseau E., Januario T., Hamman B.D., Rose C.M., Cheung T.K., Hinkle T., Soto L., Quinn C. (2022). Selective PROTAC-mediated degradation of SMARCA2 is efficacious in SMARCA4 mutant cancers. Nat. Commun..

[173] Bondeson D.P., Smith B.E., Burslem G.M., Buhimschi A.D., Hines J., Jaime-Figueroa S., Wang J., Hamman B.D., Ishchenko A., Crews C.M. (2018). Lessons in PROTAC Design from Selective Degradation with a Promiscuous Warhead. Cell Chem. Biol..

[174] Li M., Wei Y., Liu Y., Wei J., Zhou X., Duan Y., Chen S., Xue C., Zhan Y., Zheng L. (2023). BRD7 inhibits enhancer activity and expression of BIRC2 to suppress tumor growth and metastasis in nasopharyngeal carcinoma. Cell Death Dis..

[175] Liu Y., Zhao R., Wang H., Luo Y., Wang X., Niu W., Zhou Y., Wen Q., Fan S., Li X. (2016). miR-141 is involved in BRD7-mediated cell proliferation and tumor formation through suppression of the PTEN/AKT pathway in nasopharyngeal carcinoma. Cell Death Dis..

[176] Jin J., Chen F., He W., Zhao L., Lin B., Zheng D., Chen L., He H., He Q. (2023). YAP-Activated SATB2 Is a Coactivator of NRF2 That Amplifies Antioxidative Capacity and Promotes Tumor Progression in Renal Cell Carcinoma. Cancer Res..

[177] Li L., Wang L., Liu D., Zhao Y. (2023). BRD7 suppresses tumor chemosensitivity to CHK1 inhibitors by inhibiting USP1-mediated deubiquitination of CHK1. Cell Death Discov..

[178] Clark P.G., Vieira L.C., Tallant C., Fedorov O., Singleton D.C., Rogers C.M., Monteiro O.P., Bennett J.M., Baronio R., Muller S. (2015). LP99: Discovery and Synthesis of the First Selective BRD7/9 Bromodomain Inhibitor. Angew. Chem..

[179] Muller M.R., Burmeister A., Skowron M.A., Stephan A., Bremmer F., Wakileh G.A., Petzsch P., Kohrer K., Albers P., Nettersheim D. (2022). Therapeutical interference with the epigenetic landscape of germ cell tumors: A comparative drug study and new mechanistical insights. Clin. Epigenetics.

[180] Hugle M., Regenass P., Warstat R., Hau M., Schmidtkunz K., Lucas X., Wohlwend D., Einsle O., Jung M., Breit B. (2020). 4-Acyl Pyrroles as Dual BET-BRD7/9 Bromodomain Inhibitors Address BETi Insensitive Human Cancer Cell Lines. J. Med. Chem..

[181] Martin L.J., Koegl M., Bader G., Cockcroft X.L., Fedorov O., Fiegen D., Gerstberger T., Hofmann M.H., Hohmann A.F., Kessler D. (2016). Structure-Based Design of an in Vivo Active Selective BRD9 Inhibitor. J. Med. Chem..

[182] Theodoulou N.H., Bamborough P., Bannister A.J., Becher I., Bit R.A., Che K.H., Chung C.W., Dittmann A., Drewes G., Drewry D.H. (2016). Discovery of I-BRD9, a Selective Cell Active Chemical Probe for Bromodomain Containing Protein 9 Inhibition. J. Med. Chem..

[183] Zhou L., Yao Q., Li H., Chen J. (2021). Targeting BRD9 by I-BRD9 efficiently inhibits growth of acute myeloid leukemia cells. Transl Cancer Res..

[184] Zhang C., Chen L., Lou W., Su J., Huang J., Liu A., Xu Y., He H., Gao Y., Xu D. (2021). Aberrant activation of m6A demethylase FTO renders HIF2alpha(low/-) clear cell renal cell carcinoma sensitive to BRD9 inhibitors. Sci. Transl. Med..

[185] Zhu Q., Gu X., Wei W., Wu Z., Gong F., Dong X. (2023). BRD9 is an essential regulator of glycolysis that creates an epigenetic vulnerability in colon adenocarcinoma. Cancer Med..

[186] Kapoor S., Damiani E., Wang S., Dharmanand R., Tripathi C., Tovar Perez J.E., Dashwood W.M., Rajendran P., Dashwood R.H. (2022). BRD9 Inhibition by Natural Polyphenols Targets DNA Damage/Repair and Apoptosis in Human Colon Cancer Cells. Nutrients.

[187] Yang Q., Bariani M.V., Falahati A., Khosh A., Lastra R.R., Siblini H., Boyer T.G., Al-Hendy A. (2022). The Functional Role and Regulatory Mechanism of Bromodomain-Containing Protein 9 in Human Uterine Leiomyosarcoma. Cells.

[188] Barghout S.H., Mann M.K., Aman A., Yu Y., Alteen M.G., Schimmer A.D., Schapira M., Arrowsmith C.H., Barsyte-Lovejoy D. (2022). Combinatorial Anticancer Drug Screen Identifies Off-Target Effects of Epigenetic Chemical Probes. ACS Chem. Biol..

[189] Mancarella C., Morrione A., Scotlandi K. (2023). PROTAC-Based Protein Degradation as a Promising Strategy for Targeted Therapy in Sarcomas. Int. J. Mol. Sci..

[190] Remillard D., Buckley D.L., Paulk J., Brien G.L., Sonnett M., Seo H.S., Dastjerdi S., Wuhr M., Dhe-Paganon S., Armstrong S.A. (2017). Degradation of the BAF Complex Factor BRD9 by Heterobifunctional Ligands. Angew. Chem..

[191] Brien G.L., Remillard D., Shi J., Hemming M.L., Chabon J., Wynne K., Dillon E.T., Cagney G., Van Mierlo G., Baltissen M.P. (2018). Targeted degradation of BRD9 reverses oncogenic gene expression in synovial sarcoma. eLife.

[192] Kurata K., Samur M.K., Liow P., Wen K., Yamamoto L., Liu J., Morelli E., Gulla A., Tai Y.T., Qi J. (2023). BRD9 Degradation Disrupts Ribosome Biogenesis in Multiple Myeloma. Clin. Cancer Res..

[193] Zhang J., Duan H., Gui R., Wu M., Shen L., Jin Y., Pang A., Yu X., Zeng S., Zhang B. (2023). Structure-based identification of new orally bioavailable BRD9-PROTACs for treating acute myelocytic leukemia. Eur. J. Med. Chem..

[194] Zoppi V., Hughes S.J., Maniaci C., Testa A., Gmaschitz T., Wieshofer C., Koegl M., Riching K.M., Daniels D.L., Spallarossa A. (2019). Iterative Design and Optimization of Initially Inactive Proteolysis Targeting Chimeras (PROTACs) Identify VZ185 as a Potent, Fast, and Selective von Hippel-Lindau (VHL) Based Dual Degrader Probe of BRD9 and BRD7. J. Med. Chem..

[195] Ordonez-Rubiano S.C., Maschinot C.A., Wang S., Sood S., Baracaldo-Lancheros L.F., Strohmier B.P., McQuade A.J., Smith B.C., Dykhuizen E.C. (2023). Rational Design and Development of Selective BRD7 Bromodomain Inhibitors and Their Activity in Prostate Cancer. J. Med. Chem..

[196] Pan J., McKenzie Z.M., D’Avino A.R., Mashtalir N., Lareau C.A., St Pierre R., Wang L., Shilatifard A., Kadoch C. (2019). The ATPase module of mammalian SWI/SNF family complexes mediates subcomplex identity and catalytic activity-independent genomic targeting. Nat. Genet..

